# *Serratia* Secondary Metabolite Prodigiosin Inhibits *Pseudomonas aeruginosa* Biofilm Development by Producing Reactive Oxygen Species that Damage Biological Molecules

**DOI:** 10.3389/fmicb.2016.00972

**Published:** 2016-06-27

**Authors:** Önder Kimyon, Theerthankar Das, Amaye I. Ibugo, Samuel K. Kutty, Kitty K. Ho, Jan Tebben, Naresh Kumar, Mike Manefield

**Affiliations:** ^1^School of Biotechnology and Biomolecular Sciences, The University of New South WalesSydney, NSW, Australia; ^2^Department of Infectious Diseases and Immunology, Sydney Medical School, The University of SydneySydney, NSW, Australia; ^3^School of Chemistry, The University of New South WalesSydney, NSW, Australia; ^4^Ecological Chemistry, Alfred Wegener Institute for Polar and Marine Research InstituteBremerhaven, Germany

**Keywords:** prodigiosin, *Pseudomonas aeruginosa*, extracellular DNA, reactive oxygen species, biofilm inhibition

## Abstract

Prodigiosin is a heterocyclic bacterial secondary metabolite belonging to the class of tripyrrole compounds, synthesized by various types of bacteria including *Serratia* species. Prodigiosin has been the subject of intense research over the last decade for its ability to induce apoptosis in several cancer cell lines. Reports suggest that prodigiosin promotes oxidative damage to double-stranded DNA (dsDNA) in the presence of copper ions and consequently leads to inhibition of cell-cycle progression and cell death. However, prodigiosin has not been previously implicated in biofilm inhibition. In this study, the link between prodigiosin and biofilm inhibition through the production of redox active metabolites is presented. Our study showed that prodigiosin (500 μM) (extracted from *Serratia marcescens* culture) and a prodigiosin/copper(II) (100 μM each) complex have strong RNA and dsDNA cleaving properties while they have no pronounced effect on protein. Results support a role for oxidative damage to biomolecules by H_2_O_2_ and hydroxyl radical generation. Further, it was demonstrated that reactive oxygen species scavengers significantly reduced the DNA and RNA cleaving property of prodigiosin. *P. aeruginosa* cell surface hydrophobicity and biofilm integrity were significantly altered due to the cleavage of nucleic acids by prodigiosin or the prodigiosin/copper(II) complex. In addition, prodigiosin also facilitated the bactericidal activity. The ability of prodigiosinto cause nucleic acid degradation offers novel opportunities to interfere with extracellular DNA dependent bacterial biofilms.

## Introduction

Biofilm formation and persistence is controlled by the biofilm matrix or extracellular polymeric substances (EPS) which represent about 90% of the total dry mass of the biofilm (Flemming and Wingender, 2010). Biofilm matrices act as a shield thereby protecting microorganisms against protozoan grazers, chemical toxins and host immune defenses, while it allows diffusion of oxygen, nutrients and waste products through the matrix (Kokare et al., 2009; Flemming and Wingender, 2010). The biofilm matrix primarily consists of polysaccharides, proteins, lipids, nucleic acids (DNA and RNA), and metabolites. The composition of EPS can be distinct among biofilms according to the type of microorganisms and growth conditions. Polysaccharides and proteins constitute the majority (~75–90%) of EPS whereas lipids and nucleic acids constitute a minority (~1–10%; More et al., 2014). Although nucleic acids are minor component of EPS by weight, they can be a major structural component. Removal of extracellular DNA (eDNA) from EPS disrupts biofilms formed by bacterial species including *Staphylococcus epidermidis, Bacillus cereus, Neisseria meningiditis*, and *Pseudomonas aeruginosa* (Whitchurch et al., 2002; Liu et al., 2008; Vilain et al., 2009; Das et al., 2010; Lappann et al., 2010).

Bacterial biofilms usually develop as multilayers when bacteria adhere to either biotic or abiotic surfaces and also to each other or another organism (Karatan and Watnick, 2009). Development of biofilms may vary according to the type of bacterial species and environmental parameters such as pH, temperature, nutritional composition and depletion of oxygen (McDougald et al., 2012). Strategies to remove biofilms are mainly focused on induction of biofilm dispersal to reduce the drug resistance of biofilm cells. It has been shown that oxidative stress or nitrosative stress inside biofilms contributes to biofilm dispersal (Webb et al., 2003; Barraud et al., 2006). Oxidative stress results in the production of cytotoxic reactive oxygen species (ROS) including hydrogen peroxide (H_2_O_2_), superoxide (${\text{O}}_{2}^{-}$) and hydroxyl radicals (^•^OH) that damage biomacromolecules such as proteins, nucleic acids and lipids (Droge, 2003). ROS can also attack EPS, which can cause disruption of biofilm structure. ROS production has been described in the presence of different types of bactericidal antibiotics which have been linked to bacterial cell death in various species such as *Escherichia coli, S. aureus*, and *P. aeruginosa* (Kohanski et al., 2007; Jensen et al., 2014).

*P. aeruginosa* is an opportunistic pathogen which has been commonly associated with nosocomial infections (Gellatly and Hancock, 2013). Biofilm growth of *P. aeruginosa* allows bacteria to develop 1000 fold tolerance to antimicrobial agents thereby impeding the treatment of infections which result in high morbidity and mortality rates (Harmsen et al., 2010; Gellatly and Hancock, 2013). The biofilm development of *P. aeruginosa* is a five-stage cycle triggered by attachment of planktonic cells to a substratum followed by development, maturation of the biofilm matrix and dispersal of single cells, (Stoodley et al., 2002). Production of EPS during biofilm development allows bacteria to adhere tightly to neighboring bacteria and colonized surface (Stoodley et al., 2002). Factors that contribute to *P. aeruginosa* cell surface attachment and biofilm matrix development are pili, flagella, rhamnolipids, proteins, exopolysaccharides, and eDNA (Mann and Wozniak, 2012).

**Figure 1:**
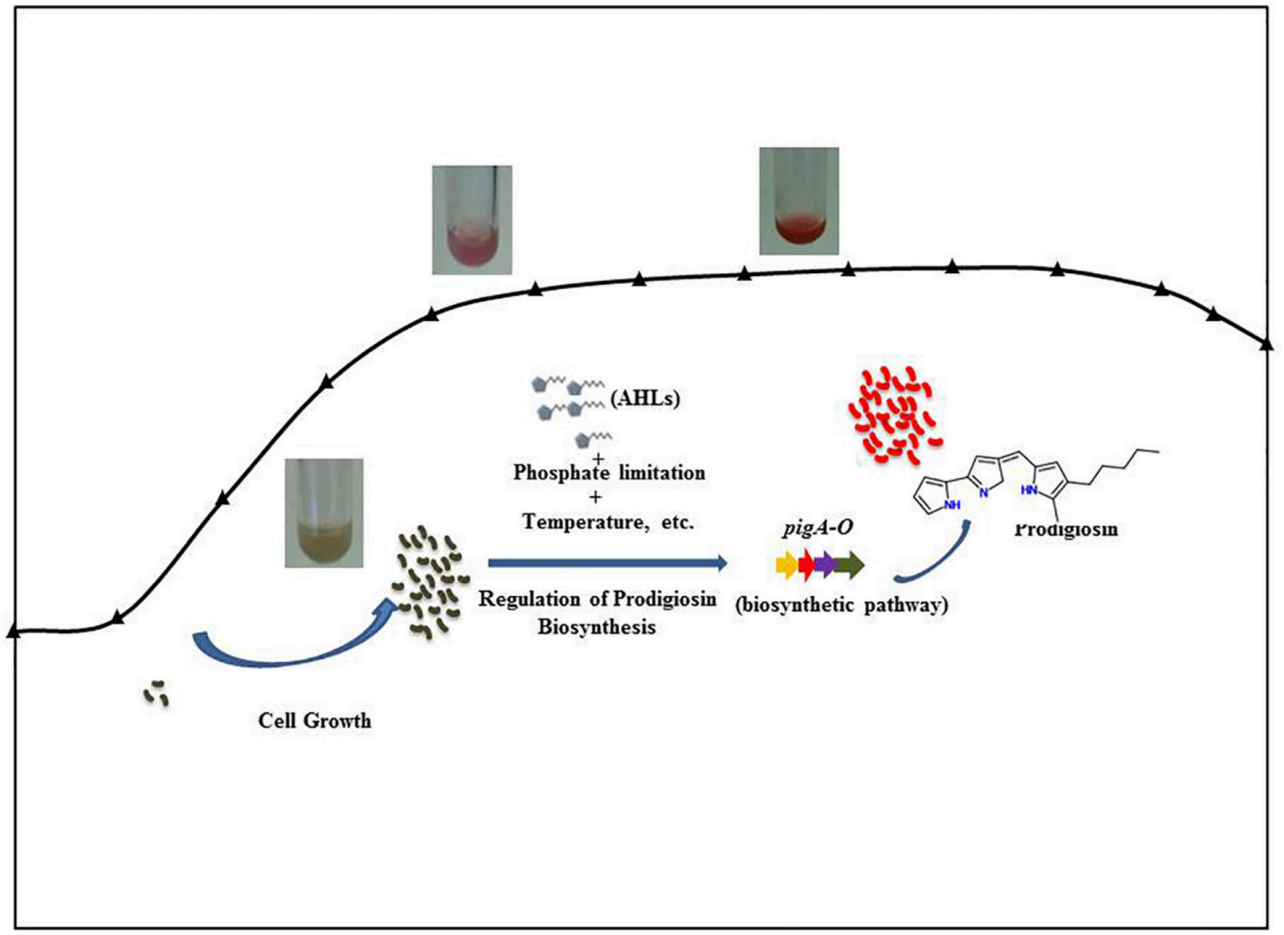
**Regulation of prodigiosin biosynthesis in *S. marcescens***.

Prodigiosin (2-methyl-3-pentyl-6-methoxyprodiginine) is a red colored heterocyclic secondary metabolite that belongs to the class of tripyrrole compounds. Prodigiosin production has been reported in *Pseudomonas, Vibrio, Hahella*, and *Serratia* species (Harris et al., 2004; Fineran et al., 2005; Jeong et al., 2005; Williamson et al., 2006). In *Serratia*, prodigiosin synthesis is mediated by QS activity, transcriptional regulators such as cyclic-adenosine-monophosphate (cAMP) receptor protein (CRP) and cyclic-di-guanosine-monophosphate (cGMP), and environmental factors such as pH and temperature through coordinating expression of the prodigiosin biosynthetic genes (*pigA-N*), (Harris et al., 2004; Fineran et al., 2005; Figure 1). The role of prodigiosin in the biology of the producing strains is not clearly understood. However, prodigiosin and related molecules have attracted attention recently due to their immunosuppressive activity and potential for cancer therapy by inducing apoptosis in several cancer cell lines (Williamson et al., 2006). Prodigiosin has been under pre-clinical trials for the treatment of pancreatic cancer (Williamson et al., 2006). Reports suggest that prodigiosin complexed with copper intercalates with DNA and promotes oxidative damage to DNA leading to inhibition of cell-cycle progression and cell death (Perez-Tomas et al., 2003; Williamson et al., 2006). Despite this interest, prodigiosin has never been considered as a biofilm control agent. In a recent study, a prodiginine type of compound, Streptorubin B (extracted from *Streptomyces sp*.), has shown to be a very potent inhibitor of biofilm formation of *S. aureus*. However, the exact biofilm inhibitory mechanism of Streptorubin B remains unknown (Suzuki et al., 2015).

To investigate the role of prodigiosin on biofilm formation of *P. aeruginosa*, biofilm cultures were treated with various concentrations of prodigiosin and prodigiosin/copper(II) complex [Prd/Cu(II)] to observe the inhibition of biofilm formation as a result of oxidative damage to biomacromolecules with a focus on nucleic acids and proteins. This study revealed that prodigiosinalone and in combination with copper [Prd/Cu(II)] significantly disrupts both biofilm establishment and pre-established biofilms of *P. aeruginosa* PA14 wild-type through an impact on nucleic acids. Evidence is presented supporting a role for production of reactive oxygen species that induce oxidative damage to DNA and RNA. These findings provide novel insights into the relationship between prodigiosin and biofilm formation that offer opportunities to interfere with bacterial biofilms.

## Materials and methods

### Bacterial strains and culture conditions

*Serratia marcescens* strain isolated from activated sludge is a wild type strain that produces prodigiosin. *S. marcescens* was routinely grown on Lysogeny broth (LB)_10_ (Bertani, 1951) agar plate at 30°C, overnight in a static condition. *P. aeruginosa* PA14 was routinely pre-cultured in LB_10_ (pH: 7.0) overnight at 37°C in a shaking incubator at 150 rpm.

### Extraction, purification, and analysis of prodigiosin from *Serratia marcescens*

*S. marcescens* cells were scraped off from the LB_10_ agar surface and prodigiosin was extracted by shaking cells with 10 ml of acidified ethanol (4% of 1 M HCl in ethanol) 3 times. Cell debris was removed by centrifugation at 13,000 rpm for 5 min. Supernatant was dried under vacuum and redissolved in methanol. Extracted pigment was purified by UHPLC (Dionex, UltiMate 3000 RSLC Systems, Thermo Scientific, USA) coupled with an Adsorbosil C18 reversed-phase column (250 × 21.20 mm, 5 μm) (Luna, Phenomenex). The flow rate was set to 10 ml/min with a solvent combination of milliQ grade water acidified with 0.1% formic acid (solvent A) and HPLC grade methanol acidified with 0.01% formic acid (solvent B). The following gradient was used for the elution: 0–1 min 100% A; 1–20 min from 100 to 0% A; 20–25 min 100% B; 25–28 min from 100 to 0% B; 28–30 min maintained at 100% A. After repeated injections, fractions containing the major peak were pooled, dried under vacuum and redissolved in either methanol or DMSO. Chemical structure of purified pigment was analyzed by ^1^H-nuclear magnetic resonance (NMR) spectroscopy and Orbitrap LTQ XL (Thermo Scientific, USA) ion trap mass spectrometer equipped with nanoelectrospray ionization source (NSI-MS) (Supplementary Figure 1). The concentration of prodigiosin was calculated by area normalization method using Chromeleon CDS software (Thermo Scientific, USA).

### Hydrogen peroxide detection assay

Hydrogen peroxide (H_2_O_2_) production from prodigiosin and copper (II) acetate (Sigma, USA) treated samples were analyzed by Amplex Red Hydrogen Peroxide assay kit (ThermoFisher Scientific, USA). Assay buffer (pH: 7.4) provided by the kit was supplemented with different concentrations of prodigiosin and copper. EDTA (10 mM) was used as metal chelator. H_2_O_2_ release was monitored over 6 h at 37°C according to manufacturer‘s instructions. Absorbance readings were taken by a multiwell plate reader (Perkin Elmer, USA). All samples were prepared in triplicates.

### DNA and RNA cleavage assay

Calf thymus DNA sodium salt (type I fibers, 42% GC content, Sigma-Aldrich, USA) was dissolved in distilled water. The concentration of double-stranded DNA (dsDNA) (~90%) present in the DNA stock solution was quantified using a broad range double stranded DNA fluorescent dye assay (Qubit, Invitrogen, USA). Total RNA was extracted from *P. aeruginosa*PA14 cultures at mid-logarithmic growth phase using RNeasy Mini Kit (QIAGEN, Germany). Prodigiosin and Prd/Cu(II) treated dsDNA (~200 ng/μl) and RNA (~120 ng/μl) samples were incubated overnight at 37°C and room temperature, respectively. Tert-butanol (2 M) and *N*-acetylcysteine (NAC) (10 mM) were used as hydroxyl radical (^•^OH) scavenger and reactive oxygen species scavenger, respectively, where it is necessary. Decrease in dsDNA concentration due to the cleaving was monitored by agarose gel electrophoresis. Gels were supplemented with GelRed (Biotium, USA) and visualized by the GeneGenius Gel Imaging System (Syngene, UK). Relative intensity of DNA bands on agarose gels were measured with ImageJ software. Decrease in RNA concentration due to the cleaving was measured with broad range RNA fluorescent dye assay (Qubit, Invitrogen, USA) according to manufacturer‘s instructions. Non-treated dsDNA and RNA were used as control. All samples were analyzed at least 3 times.

### Protein degradation assay

Bovine serum albumin (BSA) (2 μg/ml) was used as the target protein to analyse the interactions between proteins and prodigiosin. Prodigiosin and Prd/Cu(II) treated BSA samples were incubated overnight at room temperature. Tert-butanol (2 M) and *N*-acetylcysteine (NAC) (10 mM) were used as hydroxyl radical (^•^OH) scavenger and reactive oxygen species scavenger, respectively, where it is necessary. Protein concentration was measured with NanoOrange Protein Quantitation Kit (ThermoFisher, USA). Fluorescence (excitation, 470 nm; emission, 570 nm) was measured by multiwell plate reader (Ensight Plate Reader, PerkinElmer, USA). All samples were analyzed in triplicates.

### Analysis of the DNA-prodigiosin interaction

A Chirascan CD spectrophotometer (Applied Photophysics, UK) was used to investigate DNA-prodigiosin and DNA-DNase I reactions in a 1-mm path length quartz cuvette. For all experiments dsDNA concentration was kept constant (135 ng/μl) with varying prodigiosin concentrations (25, 50, 100, and 500 μM) and DNase I (Invitrogen, USA) (40 units) in 350 μl Milli-Q water were scanned from 200 to 320 nm wavelength range after an incubation for 30-min in a static condition at 25°C.

Prodigiosin binding to DNA was also assessed using a Varian Cary 100 Bio UV-Visible spectrophotometer (Varian, USA) in a 1-ml quartz cuvette on DNA, prodigiosin, and the DNA-prodigiosin mixture solution in Milli-Q water. The solutions were UV-Vis spectroscopic scanned from 200 to 800 nm wavelength.

### Crystal violet staining

*P. aeruginosa* was grown in LB_10_ overnight at 37°C with shaking at 150 r.p.m. Culture was diluted (1:20) in LB_10_ and 200 μl of aliquots were dispensed to flat bottom 96-well plate wells (Sarstedt Australia). Cultures were supplemented with varying concentrations of prodigiosin (50, 100, and 500 μM). Plate was sealed with self-adhesive microplate sealers (TopSeal-A, PerkinElmer) to allow air diffusion and to prevent condensation. Cultures were incubated overnight at 37°C with shaking at 150 r.p.m. Biofilms adhered on polystyrene substratum were quantified by crystal violet staining as described previously (O'Toole, 2011). All cultures were prepared in triplicates. Error bars represent standard deviation.

### Growth curve analysis and propidium iodide staining of *P. aeruginosa* PA14 biofilm

To analyse growth curve, *P. aeruginosa* PA14 wild-type cultures were grown in the presence of increasing concentrations of prodigiosin (20–500 μM) and copper (20–200 μM). Optical density was measured at 600 nm using multiwell plate reader (Perkin Elmer, USA).

*P. aeruginosa* PA14 biofilm was grown in 300 μl of LB_10_ in a 96-well plate supplemented with/without prodigiosin (500 μM) and Prd/Cu(II) (100 μM). Cultures were incubated overnight at 37°C in a shaking incubator at 120 rpm. Propidium iodide (PI) was used to stain nucleic acids in biofilms. PI was dissolved in distilled water at a final concentration of 6 μM. Each well was incubated with 200 μl of PI solution over 30 min at room temperature and fluorescence (535/620 nm) was measured using multiwell plate reader (Perkin Elmer, USA). Washed biofilms were treated with DNaseI and RNaseA (Qiagen, USA) over 30 min at 37°C, where it is required. All cultures were prepared in triplicates.

### Determination of *P. aeruginosa* cell surface and substratum (glass and polystyrene) hydrophobicity using contact angle measurements

Contact angle measurement on any surface determines the hydrophobicity of that surface. Bacterial cell surface hydrophobicity is one of the crucial factors that facilitate bacterial interaction (Krasowska and Sigler, 2014). In this study we investigated the modulation in *P. aeruginosa* PA14 wild-type cell surfaces hydrophobicity when subjected to prodigiosin treatment. *P. aeruginosa* PA14 wild-type suspensions were grown and harvested as mentioned above, where indicated *P. aeruginosa* PA14 wild-type were treated with 500 μM of prodigiosin or DNase I (40 units) for 120 min at 30°C in a shaking incubator (150 rpm) and subsequently washed twice with PBS (pH: 7.0) and finally resuspended in milliQ water. Contact angle measurements on bacterial cell surface and on substratum were determined as per previously published method (Das et al., 2013a). In brief, for bacterial contact angle measurements, lawns of *P. aeruginosa* PA14 wild-type were prepared by depositing bacteria from suspension on a 0.2 μm-pore-diameter filter (Nitrocellulose membrane filter, Millipore, USA) using filter unit (Millipore, USA) and by applying syringe (Becton Dickinson, USA) pressure. The filters were air dried at room temperature until contact angle of sessile water droplets reached a plateau level. For *P. aeruginosa* PA14 wild-type (untreated or prodigiosin/DNase I treated) the plateau drying time was between 25 and 40 min. Once the plateau drying level was reached, contact angles were measured with standard polar (water, formamide) and non-polar (diiodomethane) liquids using a goniometer (KSV model 200, KSV instrumentation Pvt. Ltd, Finland). Contact angle of substratum (sterile polystyrene and glass petri dishes) were determined by directly adding liquid droplet (water, formamide and diiodomethane) on substratum surface and measured using goniometer.

### Surface thermodynamical analysis of *P. aeruginosa* PA14, polystyrene and glass substratum

To conduct surface thermodynamical analysis, the measured contact angles of substratum (polystyrene and glass) and *P. aeruginosa* cell surface (before and after prodigiosin treatment) were first converted into its respective surface free energy components comprising of Lifshitz-Van der Waals (γ^*LW*^) and acid-base (γ^*AB*^) surface free energy using the Lifshitz-Van der Waals (LW)—acid-base (AB) approach as detailed previously (van Oss, 1995; Sharma and Hanumantha Rao, 2003). In brief, we provide an example of LW-AB approach equations to determine the surface energy of bacterial cell surface in which $\gamma_{bv}^{LW}$ is the LW component of the bacterial surface energy whereas, $\gamma_{lv}^{LW}$ is the LW surface free energy of the three liquids used in this study.

$$\begin{array}{l} \frac{\cos\theta +1}{2}=\frac{\sqrt{\gamma_{bv}^{LW}\;\gamma_{lv}^{LW}}}{\gamma_{lv}}+\frac{\sqrt{\gamma_{bv}^{-}\;\gamma_{lv}^{+}}}{\gamma_{lv}}+\frac{\sqrt{\gamma_{bv}^{+}\;\gamma_{lv}^{-}}}{\gamma_{lv}} \end{array}$$

Similarly, $\gamma_{lv}^{-}$ and $\gamma_{lv}^{+}$ are the surface free energy properties of the liquids whereas; $\gamma_{bv}^{-}$ and $\gamma_{bv}^{+}$ are the electron-donating and electron-accepting surface energy properties, which combined to form the acid-base (AB) component of the bacterial surface energy according to equation:

$$\begin{array}{l} \gamma_{bv}^{AB}=2\sqrt{\gamma_{bv}^{-}\;\gamma_{bv}^{+}} \end{array}$$

Since both bacterial adhesion to the substratum and bacterial aggregation takes place in an aqueous medium, the liquid surface energy components and properties for water were used in this study i.e., $\gamma_{lv}^{LW}$ = 21.8 and $\gamma_{lv}^{-}$ and $\gamma_{lv}^{+}$ = 25.5 mJ/m^2^.

The calculated surface energy components of bacteria/substratum are further used to determine the total Gibbs free energy of bacterial aggregation or adhesion (to substratum) at close contact as separated in a Lifshitz-Van der Waals (ΔG^*LW*^) and acid-base (ΔG^*AB*^) components according to equations:

$$\begin{array}{l} \begin{array}{c} \Delta G^{LW}=-2{\left(\sqrt{\gamma_{bv}^{LW}}-\sqrt{\gamma_{lv}^{LW}}\right)}^{2}\\ \Delta G^{AB}=-4\left[\left(\sqrt{\gamma_{bv}^{+}}-\sqrt{\gamma_{lv}^{+}}\right)\left(\sqrt{\gamma_{bv}^{-}}-\sqrt{\gamma_{lv}^{-}}\right)\right]\\ \Delta G^{Total}=\Delta G^{LW}+\Delta G^{AB} \end{array} \end{array}$$

### Effect of prodigiosin on *P. aeruginosa* PA14 non-established and pre-established biofilm growth

*P. aeruginosa* biofilm was grown by adding 1.5 ml of bacterial cells (OD_600_ ~0.600) suspended in fresh LB_10_ (pH: 7.0) medium into both 35 × 10 mm polystyrene and 60 x 15 mm glass petri dishes and incubated over 24 h at 37°C in a shaking incubator at 120 rpm. Where indicated the biofilm were also grown in presence of prodigiosin (100 and 500 μM) and copper (50 and 100 μM). After 24 h, the biofilm growth medium was discarded and the biofilms were gently rinsed 3 times with PBS (pH: 7.0) in order to remove any planktonic bacterial cells. The remaining substratum adhered biofilms were then stained with Live/dead stain (Invitrogen, Oregon, USA) for 20 min in the dark and then subjected to confocal laser scanning microscopic (CLSM) (Olympus FV1200) for imaging using laser setting. Image J software analysis program was employed to get 3 dimensional images and quantify biofilm thickness, biovolume, percentage (%) of live and dead biofilm and % substratum coverage. To investigate the effect of prodigiosin on pre-established biofilm, *P. aeruginosa* wild-type biofilm was grown for 24 h using the protocol mentioned above. After 24 h, cultures were supplemented with prodigiosin (100 and 500 μM) and copper (50 and 100 μM) and incubated for another 24 h at 37°C with shaking at 120 rpm. After 24 h incubation, the biofilms were gently rinsed 3 times with PBS (pH: 7.0) followed by live/dead staining, confocal microscopic imaging and quantification as mentioned above. Control cultures were prepared with no prodigiosin. DNAse I (40 units) treated cultures were used as positive controls. All cultures were repeated at least 3 times.

### Statistical analysis

GraphPad Prism 5 software was used to apply Students *T*-tests where necessary to determine the significant differences observed in the assays. *P* value lower than 0.01(*P* < 0.01) indicated significant differences.

## Results

### Prodigiosin cleaves dsDNA and RNA through reactive oxygen species production

#### H_2_O_2_ production by prodigiosin and Prd/Cu(II)

**Figure 2:**
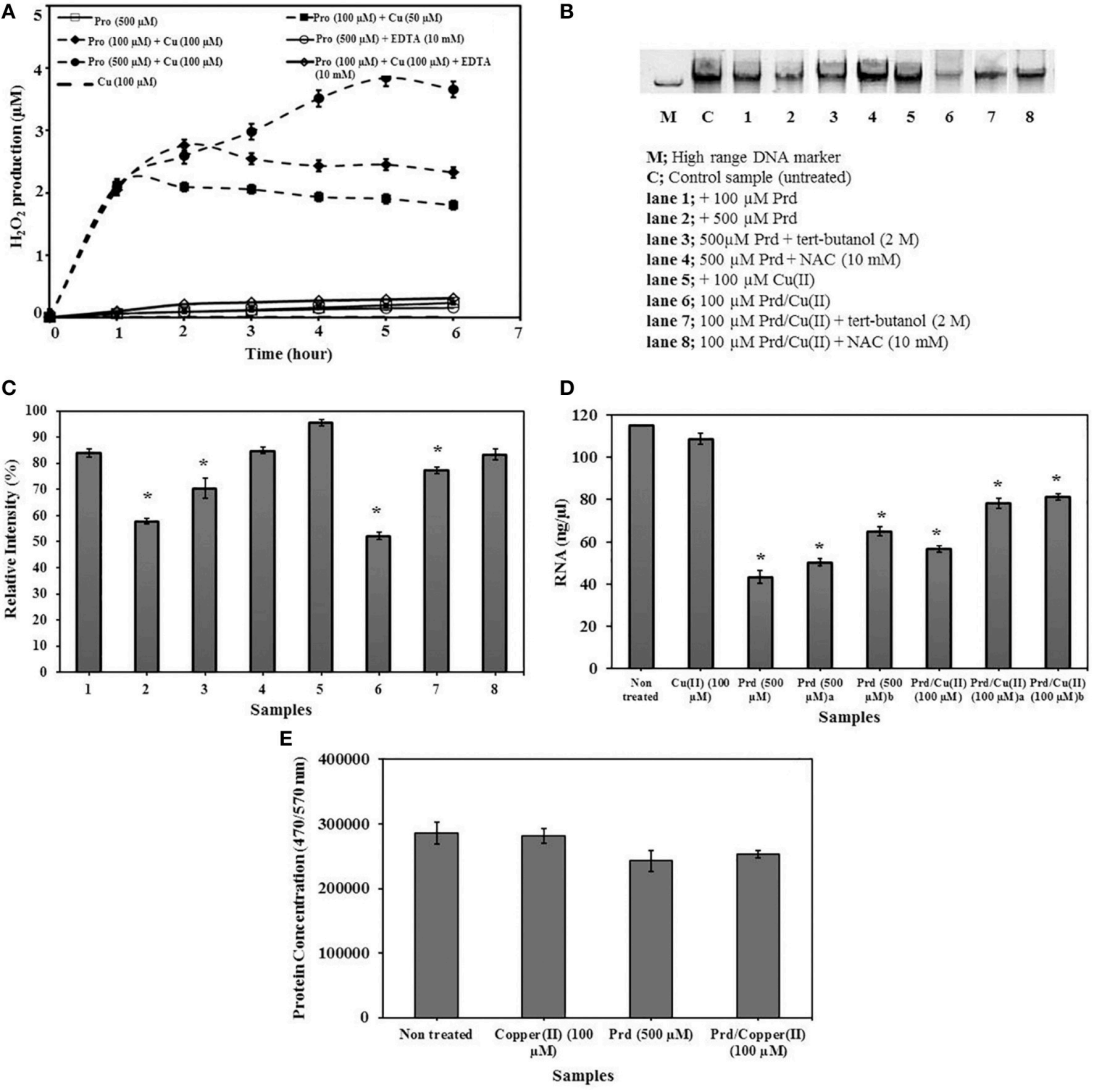
**Prodigiosin mediated H_2_O_2_ production and effect of prodigiosin on nucleic acids and proteins. (A)** H_2_O_2_ detection assay of prodigiosin (500 μM), copper (100 μM), increasing concentrations of prodigiosin/copper complex and EDTA (10 mM) treated samples. **(B)** Agarose gel electrophoresis shows the cleaving of DNA. **(C)** Relative intensities of DNA bands on agarose gel electrophoresis were quantified by ImageJ and % intensities of each band were calculated. **(D)** Total RNA of *P. aeruginosa* PA14 was treated with various concentrations of prodigiosin and prodigiosin/copper complex and concentrations of RNA were quantified by Qubit fluorometer. **(E)** BSA (2 μg/ml) was treated with copper (II) (100 μM), prodigiosin (500 μM), prodigoisin/copper complex (100 μM) and concentrations were quantified by NanoOrange protein quantitation assay. Error bars represents standard deviations from the mean (*n* = 3). Asterisks indicate the significant differences in comparison to control samples (*P* < 0.01). ^a^Tert-Butanol (2 M) ^b^NAC (10 mM).

It has been reported that Prd/Cu(II) cleaves dsDNA through facilitated prodigiosin oxidation which converts O_2_ into H_2_O_2_. No H_2_O_2_ production was reported in the absence of copper(II) at low prodigiosin concentrations (≤40 μM) (Melvin et al., 2000). A H_2_O_2_ detection assay was used to measure prodigiosin and Prd/Cu(II) mediated H_2_O_2_ production (Figure 2A). In conflict with previously published work, both prodigiosin and Prd/Cu(II) produced H_2_O_2_ from 0.3 to 4 μM over 6 h. H_2_O_2_ production was significantly (*P* < 0.01) elevated through increasing concentrations of Prd/Cu(II) and the highest H_2_O_2_ production (2–4 μM) was observed in Prd (500 μM)/Cu(II) (100 μM) treated samples. H_2_O_2_ production was 10 times lower in the absence of copper remaining at 0.3 μM in prodigiosin (500 μM) only samples. Cu(II) (100 μM) alone produced no H_2_O_2_. H_2_O_2_ production by Prd/Cu(II) samples were reduced to 0.3 μM through supplementation of EDTA (10 mM), a known metal chelator. EDTA had no impact on prodigiosin mediated H_2_O_2_ production, indicating that copper facilitates prodigiosin mediated H_2_O_2_ production.

#### Prodigiosin and Prd/Cu(II) mediated dsDNA cleaving

The effect of prodigiosin and Prd/Cu(II) on EPS components were analyzed with a focus on DNA, RNA and proteins. The impact of ROS on EPS has been extensively investigated previously and is not further examined here (Duan and Kasper, 2011). A DNA integrity assay was used to examine the effect of prodigiosin on dsDNA. All samples were monitored by agarose gel electrophoresis (Figure 2B). Relative intensities of DNA bands were significantly (*P* < 0.01) reduced in the presence of 500 μM prodigiosin and 100 μM Prd/Cu(II) (57.9 and 52.2%, respectively) (column 1, 6) in comparison to untreated control DNA samples (100.0%) (Figure 2C). Previous reports suggested that only H_2_O_2_ molecules participate in Prd/Cu(II) mediated dsDNA cleavage. To analyse the role of ROS molecules, prodigiosin (500 μM) and Prd/Cu(II) (100 μM), treated samples were supplemented with tert-butanol (2 M) as ^•^OH scavenger and *N*-acetylcysteine (NAC) (10 mM) as ROS scavenger. In contrast to reported data, supplementation of tert-butanol decreased DNA degradation in both prodigiosin (500 μM) and Prd/Cu(II) (100 μM) treated samples (loss to 70.4 and 77.4%, respectively) indicating that ^•^OH molecules also participate in prodigiosin mediated dsDNA cleavage. Additional data showed decreased DNA degradation in prodigiosin (500 μM) and Prd/Cu(II) (100 μM) treated samples with loss to 84.9 and 83.5% (column 4, 8), respectively, in the presence of NAC (10 mM) suggesting that removal of ROS molecules, most importantly H_2_O_2_, substantially inhibits dsDNA cleavage.

#### Prodigiosin and Prd/Cu(II) mediated RNA cleaving

Total RNA was extracted from *P. aeruginosa* PA14 to use in prodigiosin mediated RNA cleavage assays. RNA concentrations of all analyzed samples were measured with a fluorometer (Figure 2D). The RNA concentration in untreated controls was 115 ng/μl and this was reduced to 43 ng/μl and 56.5 ng/μl in samples treated with prodigiosin (500 μM) and Prd/Cu(II) (100 μM), respectively. Supplementation with tert-butanol partially protected the RNA (50.4 ng/μl) in the presence of prodigiosin (500 μM) while RNA remained at significantly higher concentrations (78.2 ng/μl) in the presence of Prd/Cu(II) (100 μM). Similar to dsDNA cleavage, NAC (10 mM) inhibited RNA cleavage of both prodigiosin and Prd/Cu(II) treated samples and RNA concentrations remained at 65 ng/μl and 81.3 ng/μl, respectively. The strong inhibition of dsDNA and RNA cleavage reactions by tert-butanol treatment suggests copper(II) promotes production of ^•^OH molecules in the presence of prodigiosin.

#### Effect of prodigiosin and Prd/Cu(II) on proteins

In addition to dsDNA and RNA, the effect of prodigiosin and Prd/Cu(II) was also analyzed on proteins. BSA (2 μg/ml) was used as a target macromolecule in protein degradation assays treated with 500 μM prodigiosin or 100 μM Prd/Cu(II) (Figure 2E). Prodigiosin in the presence and absence of copper failed to degrade BSA suggesting that prodigiosin and Prd/Cu(II) could be more effective on removal of nucleic acids instead of proteins in a given EPS.

### Prodigiosin exhibits similar patterns as DNase I in dsDNA cleavage

**Figure 3:**
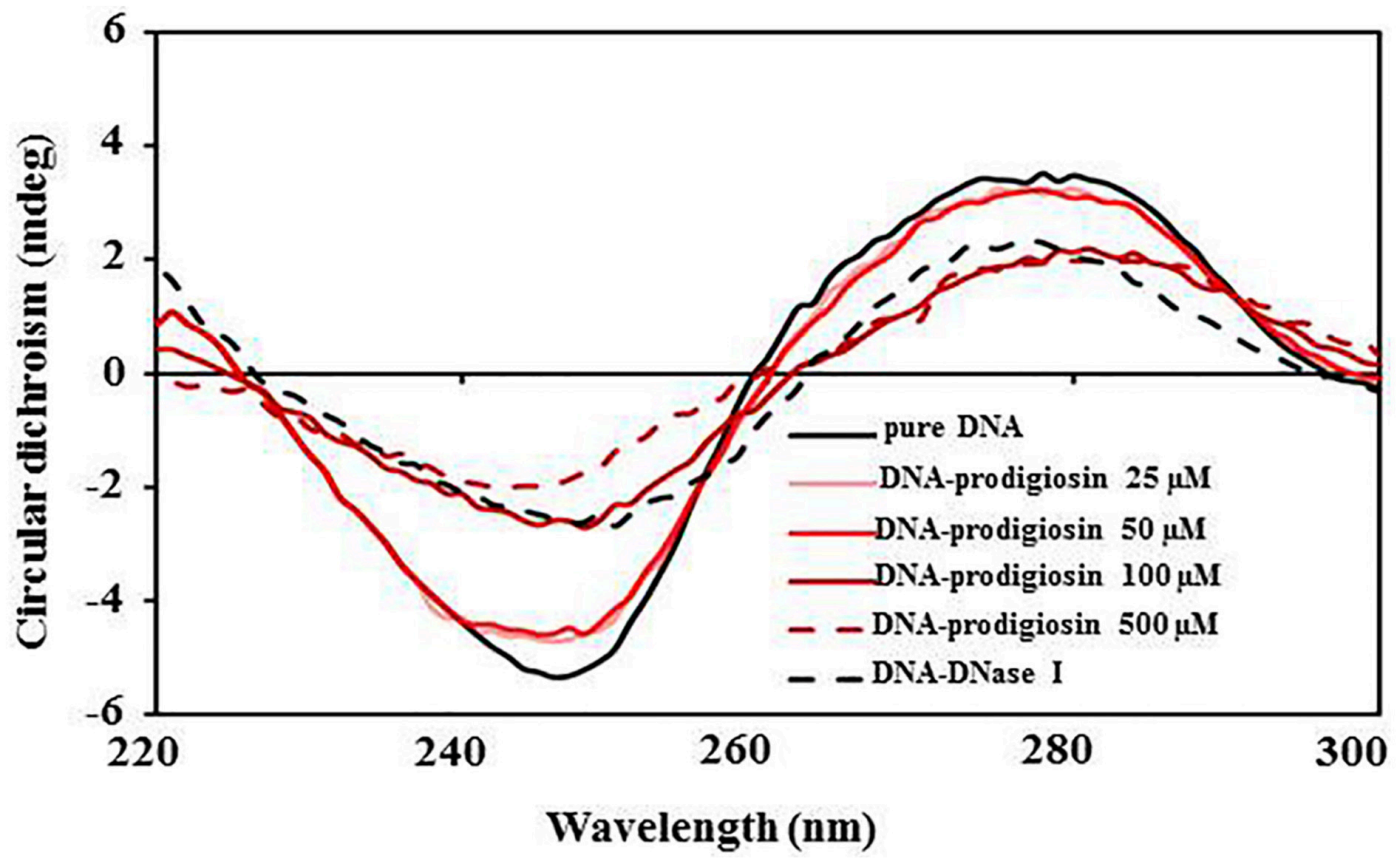
**Prodigiosin mediated cleaving of DNA**. Circular dichroism spectra of pure DNA, DNA-prodigiosin and DNA-DNase I mixture in distilled water recorded at 25°Cis shown. The change in mdeg of DNA spectra at wavelengths ~247 and 277 nm is due to binding and consequently cleaving of DNA with increasing concentrations of prodigiosin and DNase I.

In conflict with previous reports, results presented in this study suggest that prodigiosin itself cleaves dsDNA in the absence of copper(II) ions. To understand the interactions between prodigiosin and dsDNA, conformational differences of dsDNA in the presence of prodigiosin were analyzed by circular dichroism (CD). Comparison of CD spectra of DNA, DNA-prodigiosin and DNA-DNase I mixtures confirmed that prodigiosin mediates DNA cleavage (Figure 3). As the concentration of prodigiosin increases (25, 50, 100, and 500 μM), the peak intensity of characteristic DNA peaks at 247 and 277 nm changed whilst no shift in the control DNA peaks were observed. The changes in the DNA peak of DNA-prodigiosin mixtures was similar to DNA-DNase I peaks suggesting prodigiosin, like DNase I, cleaves the DNA polymer into smaller (di or tri) nucleotides.

### Effect of prodigiosin on *P. aeruginosa* PA14 cell surface hydrophobicity, surface energies and Gibbs free energy of aggregation and adhesion

#### Effect of prodigiosin on *P. aeruginosa* PA14 cell surface hydrophobicity

Previous studies showed removal of eDNA from *P. aeruginosa* significantly alters bacterial cell surface hydrophobicity (Das et al., 2013a). The influence of prodigiosin on bacterial cell surface hydrophobicity was therefore tested. Table 1 shows the contact angle values of polystyrene and glass substratum and *P. aeruginosa* PA14 treated with/without prodigiosin (500 μM). Untreated PA14 has a water contact angle of about 49 degree, which is significantly (*P* < 0.01) higher than prodigiosin treated cells at about 37 degree, consistent with the hypothesis that prodigiosin cleaves eDNA released by *P. aeruginosa* PA14. Prodigiosin itself is a hydrophobic compound (Rosenberg et al., 1986) which has a water contact angle of about 64 degree on glass substratum, so the decrease in hydrophobicity of prodigiosin treated cells indicates the observed effect was not a direct influence of prodigiosin absorbtion to cells. The subsequent surface energy components measured based on contact angle values remained unaffected between PA14 only and PA14 treated with prodigiosin for Lifshitz-Van der Waals (γ^*LW*^). Whereas, the electron donating (γ^−^) and electron accepting (γ^+^) factors of the acid-base (γ^*AB*^) component was significantly (*P* < 0.01) different between the PA14 only and PA14treated with prodigiosin.

**Table 1:** **Effect of prodigiosin on *P. aeruginosa* PA14 wild-type and polystyrene and glass substratum cell surface hydrophobicity (contact angle) and surface energies**.

	**Contact angle (θ) − degrees (°)**			**Surface energy (mJ/m^2^)**			
	**Water**	**Formamide**	**Diiodomethane**	**γ*LW***	**γ^−^**	**γ^+^**	**γ*AB***
***P. aeruginosa*** **PA14 WILD-TYPE**							
Non treated	**49** ± **4**	**39** ± **2**	**50** ± **1**	**34** ± **1**	**30** ± **4**	**1** ± **0.1**	**12** ± **1**
Prodigiosin treated	37 ± 4	52 ± 5	37 ± 2	41 ± 1	58 ± 8	0 ± 0	0 ± 0
**POLYSTYRENE SUBSTRATUM**							
	79 ± 5	72 ± 2	27 ± 4	45 ± 1	16 ± 7	0 ± 0	0 ± 0
**GLASS SUBSTRATUM**							
	13 ± 4	11 ± 5	44 ± 8	38 ± 4	53 ± 4	2 ± 1	19 ± 6

*Contact angle measurements of P. aeruginosa PA14 wild-type (before and after treatment with prodigiosin) and polystyrene and glass substratum using polar (water and formamide) and non-polar (diiodomethane) liquids. Contact angles values used to calculate bacteria and polystyrene and glass substratum, surface free energy components: Lifshitz-Van der Waals (γ^LW^), electron-donating (γ^−^) and electron-accepting (γ^+^) and acid-base (γ^AB^). ± represents standard deviations from the mean (n = 3). Bold indicate statistically significant (P < 0.01) differences in contact angle and surface energy components in comparison to prodigiosin treated wild-type*.

#### Gibbs free energy of *P. aeruginosa* aggregation and adhesion

**Figure 4:**
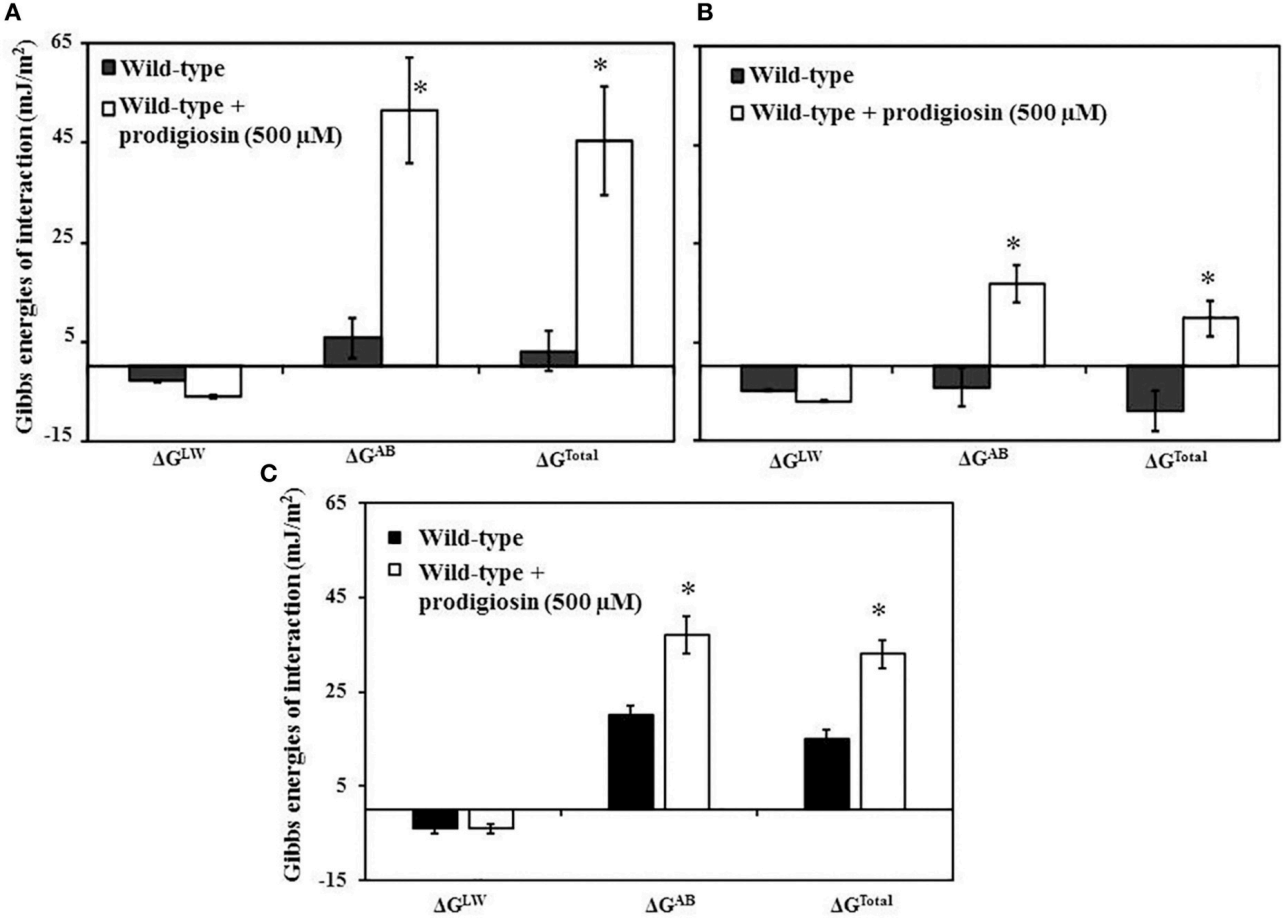
**Components of Gibbs free energy of interactions: Lifshitz-Van der Waals (ΔG^*LW*^), acid-base (ΔG^*AB*^) and total interfacial free energy (ΔG^*Total*^) of *P. aeruginosa* PA14 (A) aggregation and adhesion to (B) polystyrene and (C) glass substratum before and after incubation/treatment with prodigiosin (500 μM)**. Error bars represents standard deviations from the mean (*n* = 3). Asterisks indicate the differences in the free energy of interaction (aggregation and adhesion) in comparison to prodigiosin treated PA14 are statistically significant (*P* < 0.01).

Figure 4 shows Gibbs free energy (ΔG) of interaction between *P. aeruginosa* cell-to-cell (aggregation) and adhesion to polystyrene and glass substratum. In both aggregation (Figure 4A) and adhesion (Figure 4B) the Lifshitz-Van der Waals interaction energies (ΔG^*LW*^) were attractive regardless of prodigiosin treatment. On the other hand, acid-base (ΔG^*AB*^) and total interaction (ΔG^*Total*^) energies were significantly (*P* < 0.01) varied between PA14 only and PA14 treated with 500 μM prodigiosin. For aggregation, non-treated PA14 showed very low positive values for both acid-base (6 mJ/m^2^) and total Gibbs free energy (3 mJ/m^2^) and significantly (*P* < 0.01) higher up to 9–15-fold increase in positive values for both acid-base (52 mJ/m^2^) and total (45 mJ/m^2^) for prodigiosin treated PA14 (Figure 4A).

For adhesion to polystyrene the non-treated PA14 showed attractive interactions i.e., negative value for both acid-base (−4 mJ/m^2^) and total (−9 mJ/m^2^) and non-attractive interaction energies i.e., positive values for both acid-base (17 mJ/m^2^) and total (10 mJ/m^2^) in case of prodigiosin treated PA14 (Figure 4B). For glass the LW is negative regardless of prodigiosin treatment but the acid-base and total Gibbs adhesion energy is positive i.e., ΔG^*Total*^ = 15 mJ/m^2^ (untreated) and 35 mJ/m^2^ (Prodigiosin treated), indicating that prodigiosinmakes colonization of glass substratum unfavorable (Figure 4C). In short, surface thermodynamics results suggest that prodigiosin impairs aggregation and adhesion (to surfaces) of *P. aeruginosa* PA14.

### Prodigiosin disrupts non-established and pre-established biofilm formation of *P. aeruginosa* PA14

**Figure 5:**
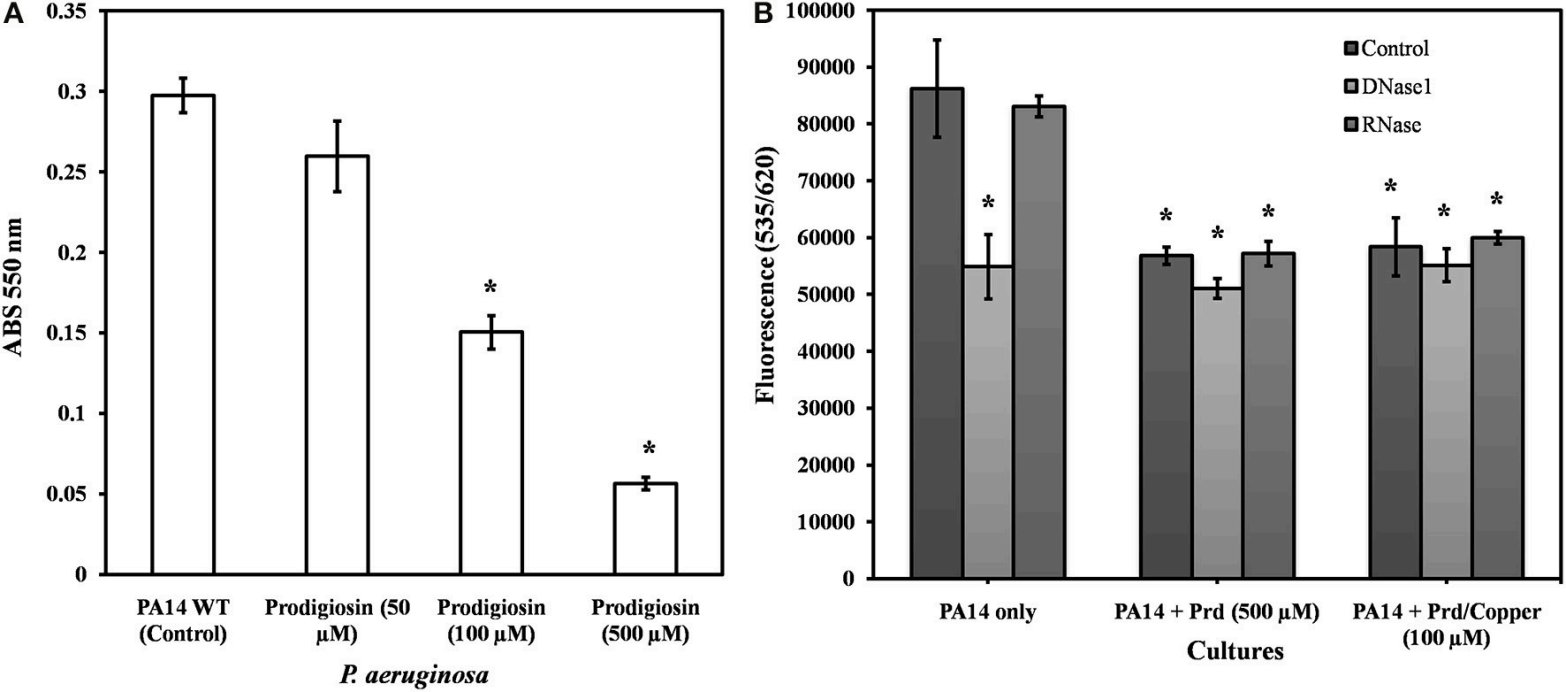
**Quantification of total biomass, eDNA and total RNA of biofilms developed by *P*. *aeruginosa* PA14 biofilms. (A)** Crystal violet staining of total biomass developed by *P. aeruginosa* PA14, **(B)** Propidium iodide staining of nucleic acids of biofilms developed by *P. aeruginosa* PA14 in LB_10_ in the presence and absence of prodigiosin (500 μM) and prodigiosin/Cu(II) (100 μM). Washed biofilms were treated with DNase I (40 units) and RNase A (20 units) over 30 min at 37°C. Error bars represents standard deviations from the mean (*n* = 3). Asterisks indicate the significant differences in comparison to control samples (*P* < 0.01).

To analyse the impact of prodigiosin on total biomass within the biofilm matrix of *P. aeruginosa*, cultures were grown on polystyrene substratum in the presence of 50, 100, and 500 μM prodigiosin (Figure 5A). Washed biofilms were stained with crystal violet to quantify total biomass. Initial observations on biofilm formation have shown that prodigiosin significantly reduces total biomass at 500 μM (~70%) and 100 μM (~35%) whereas no significant effect was observed on biofilm formation below 100 μM prodigiosin.

In order to test the effect of prodigiosin on eDNA and total RNA within the biofilm matrix of *P. aeruginosa* PA14, cultures were grown on polystyrene substratum in the presence of 500 μM prodigiosin or 100 μM Prd/Cu(II) (Figure 5B). Washed biofilms were stained with propidium iodide (PI) which specifically stains extracellular nucleic acids (Suzuki et al., 1997). Initial observations on biofilm formation revealed that nucleic acid molecules were significantly (*P* < 0.01) reduced in the biofilm matrix of *P. aeruginosa* PA14 with ~35% at 500 μM prodigiosin and ~32% at 100 μM Prd/Cu(II) treated cultures. To understand the relative roles of eDNA and RNA in the biofilm matrix, washed biofilms were treated with DNase I and RNase A, separately. Nucleic acid concentration of DNase I treated biofilms was significantly (*P* < 0.01) reduced (~36%) whereas no change was observed in RNase A treated biofilms. These results indicate that the majority of nucleic acids within EPS are composed of eDNA. DNase I and RNase A had no effect on nucleic acid concentrations of prodigiosin or Prd/Cu(II) treated biofilms.

To further analyse the effect of prodigiosin on biofilm formation of *P. aeruginosa* PA14, cultures were grown on either glass or polystyrene substratum in the presence of increasing concentrations of prodigiosin ranging from 100 to 500 μM. The biofilms of *P. aeruginosa* PA14 were similar on glass and polystyrene including non-established and pre-established biofilms based on thickness, surface coverage and biovolume.

**Figure 6:**
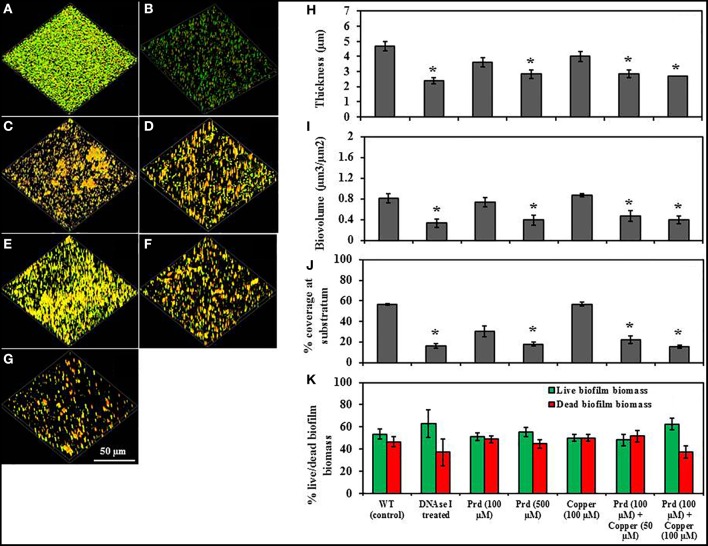
**Effect of prodigiosin and prodigiosin/Cu(II) on non-established *P*. *aeruginosa* PA14 biofilms grown on glass substratum**. All cultures were grown in LB_10_ at 37°C with shaking at 100 rpm. **(A)** Control culture (untreated), **(B)** + DNAse I, **(C)** + 100 μM prodigiosin, **(D)** + 500 μM prodigiosin, **(E)** + 100 μM Cu(II), **(F)** + 100 μM prodigiosin + 50 μM Cu(II), **(G)** + 100 μM prodigiosin + 100 μM Cu(II). Image J software was used to quantify, **(H)** biofilm thickness (μm), **(I)** biovolume (μm^3^/μm^2^), **(J)** % surface coverage at substratum and **(K)** % live/dead biofilm biomass. Error bars represents standard deviations from the mean (*n* = 3). Asterisks indicate the significant differences in comparison to control samples (*P* < 0.01).

**Figure 7:**
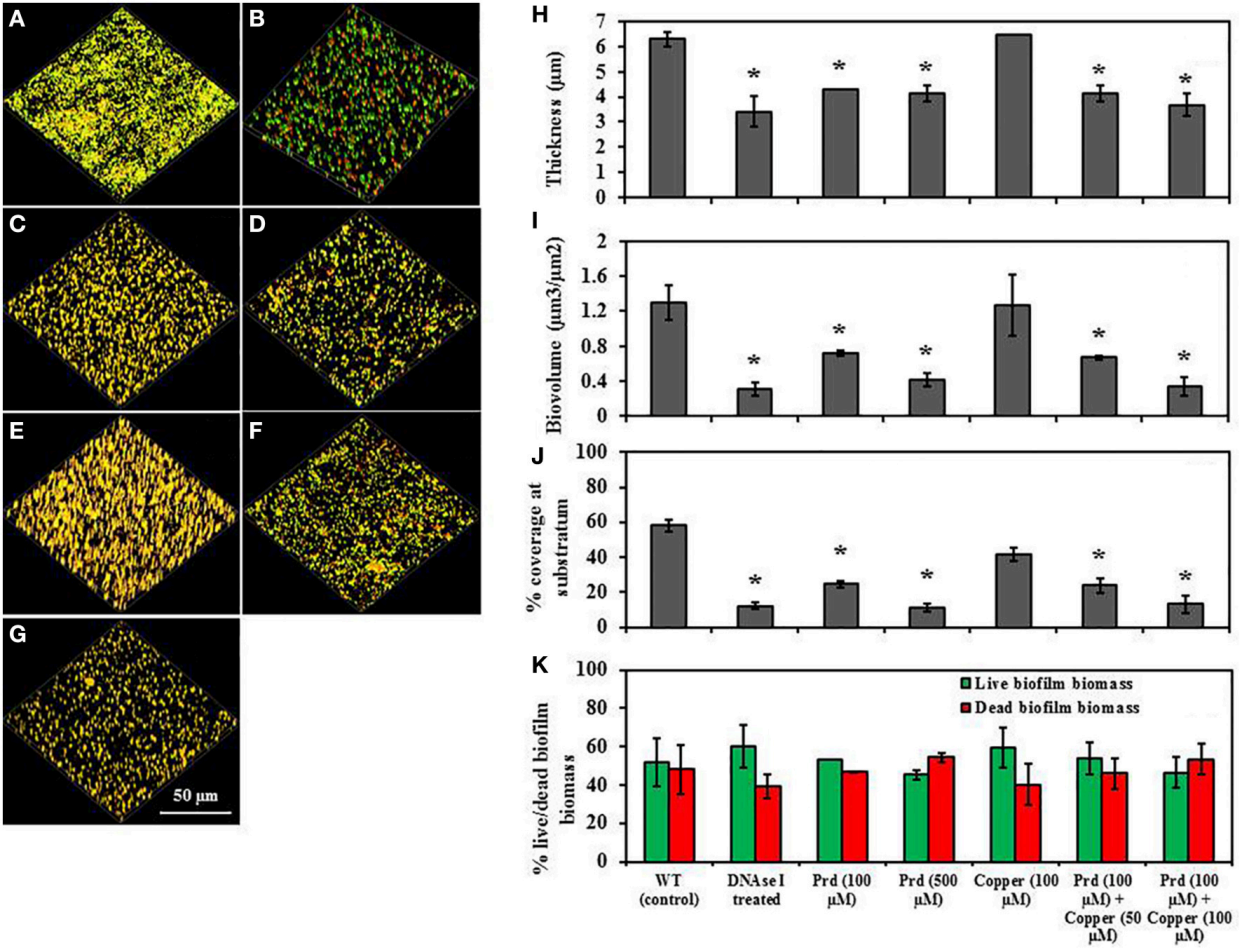
**Effect of prodigiosin and prodigiosin/Cu(II) on pre-established *P*. *aeruginosa* PA14 biofilms grown on glass substratum**. All cultures were grown in LB_10_ at 37°C with shaking at 100 rpm. **(A)** Control culture (untreated), **(B)** + DNAse I, **(C)** + 100 μM prodigiosin, **(D)** + 500 μM prodigiosin, **(E)** + 100 μM Cu(II), **(F)** + 100 μM prodigiosin + 50 μM Cu(II), **(G)** + 100 μM prodigiosin + 100 μM Cu(II). Image J software was used to quantify, **(H)** biofilm thickness (μm), **(I)** biovolume (μm^3^/μm^2^), **(J)** % surface coverage at substratum and **(K)** % live/dead biofilm biomass. Error bars represents standard deviations from the mean (*n* = 3). Asterisks indicate the significant differences in comparison to control samples (*P* < 0.01).

Figure 6 shows the effect of prodigiosin on non-established biofilm formation of *P. aeruginosa* PA14 on glass. The untreated control developed biofilms with higher thickness (4.7 μm), biovolume (0.8 μm^3^/μm^2^) and surface coverage (56.3%) (Figures 6A,H–J) in comparison to biofilms grown in the presence of DNase I or prodigiosin (Figures 6B–D,H–J). Biofilm formation was significantly disrupted by treatment with DNase I which cleaves eDNA within the biofilm matrix, with significant (*P* < 0.01) decreases in its biofilm thickness (2.4 μm), biovolume (0.3 μm^3^/μm^2^) and surface coverage (30.2%) observed (Figures 6B,H). As the prodigiosin concentration increased, biofilm development was impaired showing significantly (*P* < 0.01) reduced biofilm formation at 500 μM prodigiosin which was effective in reducing the biofilm thickness (2.8 μm), biovolume (0.4 μm^3^/μm^2^) and surface coverage (18.0%) (Figures 6D,H). Furthermore, the effect of prodigiosin (100 μM) on biofilm formation of *P. aeruginosa* PA14 was more pronounced in the presence of copper while copper itself (100 μM) showed no effect on biofilm development (Figures 6E–J). Similar results were observed on pre-established biofilms of *P. aeruginosa* PA14 in the presence and absence of prodigiosin and copper/prodigiosin (Figure 7). These findings reveal that prodigiosin (500 μM) and copper (100 μM)/prodigiosin (100 μM) complex can remove significant biovolume and transform larger aggregates in biofilms into smaller aggregates.

**Figure 8:**
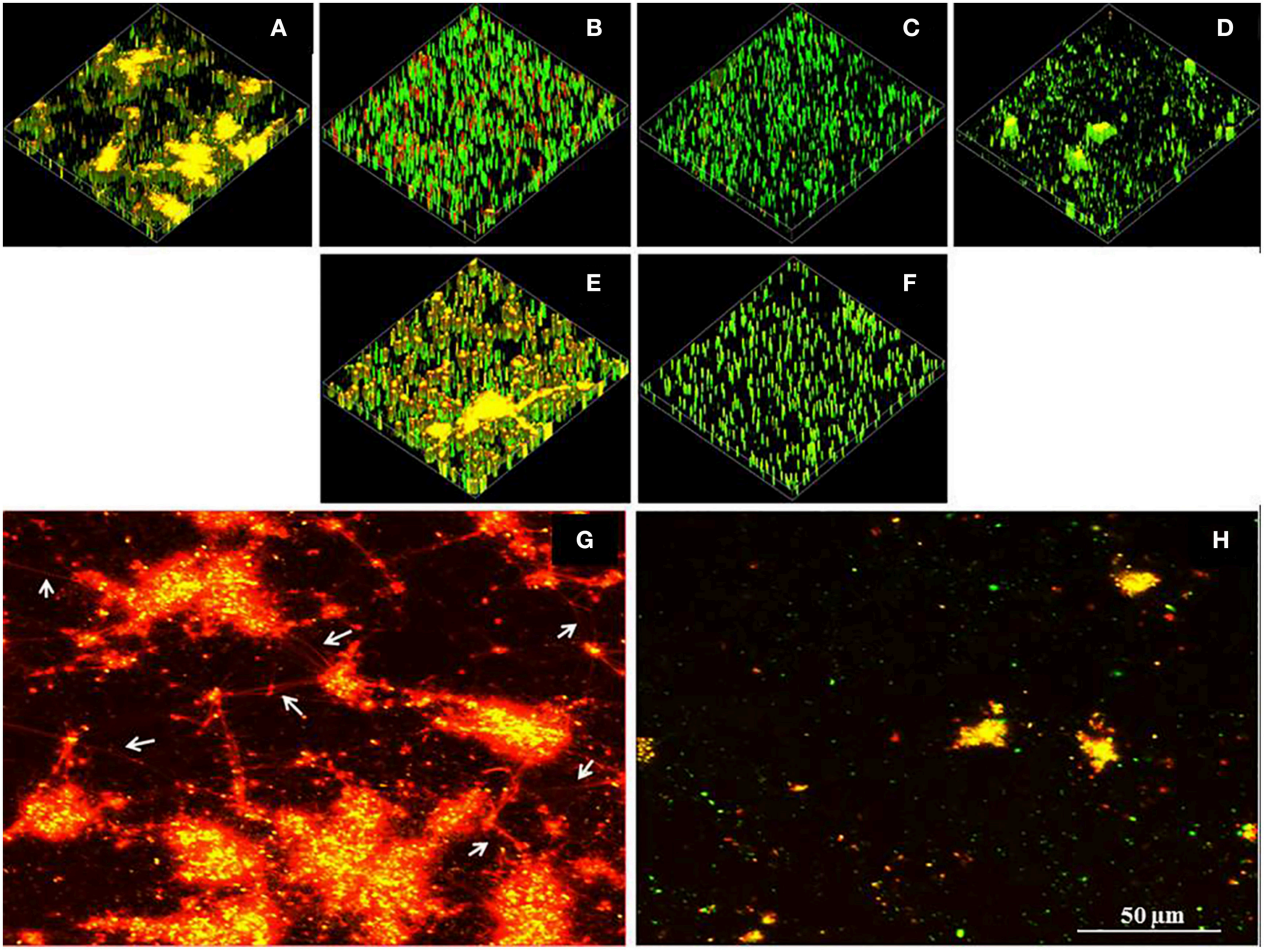
**Effect of prodigiosin on *P. aeruginosa* PA14 biofilms grown on polystyrene substratum**. All cultures were grown in LB_10_ at 37°C with shaking at 100 rpm. Non-established biofilms; **(A)** control culture (untreated), **(B)** + 100 μM prodigiosin, **(C)** + 200 μM prodigiosin and **(D)** + 500 μM prodigiosin. Pre-established biofilms; **(E)** control culture (untreated), **(F)** + 500 μM prodigiosin. Visualization of eDNA strands in **(G)** non-established biofilm control culture (pointed by arrows) and **(H)** non-established biofilm culture treated with 500 μM prodigiosin.

The impact of prodigiosin on biofilm formation was consistent on polystyrene substratum (Figures 8A–F, Supplementary Table 1). Figures 8A,G show non-treated control biofilms with clear visualization of network like structures that linked the whole biofilm. Previous research (Bockelmann et al., 2006) showed eDNA forming network like structures that integrate biofilms. Biofilm formation was disrupted in prodigiosin (500 μM) treated culture through removal (cleavage) of eDNA strands (Figures 8D,H).

### Effect of prodigiosin on *P. aeruginosa* planktonic culture

To analyse the impact of prodigiosin on growth rate of *P. aeruginosa* PA14, bacterial cultures were treated with increasing concentrations of prodigiosin ranging from 20 to 500 μM. The early logarithmic growth rate of *P. aeruginosa* was significantly (*P* < 0.01) reduced at all prodigiosin concentrations from 4 to 20 h incubation time while the bacterial cell densities of all prodigiosin treated cultures reached a similar optical density after 20 h (Supplementary Figure 2). Similar results were observed in Prd/Cu(II) treated cultures excluding 200 μM Prd/Cu(II) treated culture which showed reduced optical density after 20 h.

## Discussion

The resistance of bacteria to antimicrobial agents is an increasingly pressing public health concern. Biofilms represent evolutionary hotspots where selection for antimicrobial resistance is intense, necessitating novel approaches to control biofilms. One of the major reasons that preclude the disruption of biofilms is obstacles to the penetration of antibacterial agents into the base of biofilms to induce removal of bacterial cells (Stewart and Costerton, 2001; Kokare et al., 2009). In a biofilm, bacterial cells are mostly encased or shielded by a thick and well-integrated biofilm matrix. Extracellular DNA (eDNA) integrates the biofilm matrix of many bacterial species including *P. aeruginosa*. It binds biomolecules, cations and antibiotics and increases bacterial adhesion and aggregation strength (Das et al., 2011, 2013b, 2014; Chiang et al., 2013).

In this study, the potential of the bacterial secondary metabolite prodigiosin in the disruption of *P. aeruginosa* PA14 biofilms has been described. Both biofilm development and the integrity of mature biofilms was shown to be disrupted through application of prodigiosin alone or with a combination of prodigiosin and copper in the mid micromolar range. The effects were comparable on polystyrene and glass surfaces. Prodigiosin and copper are both involved in ROS production and it is reasonable to expect that this was the root cause of the biofilm disruption. Growth curve data of *P. aeruginosa* PA14 suggested that ROS production from prodigiosin had a significant impact on cell proliferation. Whilst this is likely to explain the inhibitory impact of prodigiosin on biofilm development, it does not explain the removal of mature biofilms. Note also that *P. aeruginosa* could ultimately overcome the growth inhibitory effects, likely by virtue of ROS catalyzing enzymes such as catalase and peroxidase (Elkins et al., 1999; Somprasong et al., 2012).

Experiments were performed to assess whether prodigiosin interfered with biofilm matrix components. Whilst RNA was heavily impacted evidence presented suggests RNA does not play a major role in biofilm development or integrity. Protein degradation assays revealed prodigiosin had no effect on BSA. The oxidation of BSA has previously been investigated using different oxidants and free radical formation has been shown to induce BSA oxidation through the Fenton reaction at low millimolar concentrations of H_2_O_2_ (Baron et al., 2006). The amount of H_2_O_2_ produced by oxidation of prodigiosin in this study was at low micromolar concentrations. Bacteria produce several types of exopolysaccharides that contribute to biofilm development and these polysaccharides can be diverse even between closely related species (Flemming and Wingender, 2010). *P. aeruginosa* produces at least three distinct exopolysaccharides (alginate, Pel, and Psl), therefore, the impact of prodigiosin on exopolysaccharides will be examined in a future study with a more comprehensive experimental approach (Flemming and Wingender, 2010).

Prodigiosin was shown to have a dose dependent effect on the integrity of dsDNA. DNA degradation is attributed to the interactions of dsDNA with hydrogen peroxide (H_2_O_2_) produced by prodigiosin and copper (Park et al., 2003). However, DNA cleavage assays revealed here that high concentration of prodigiosin can also result in dsDNA cleavage in the absence of copper. Furthermore, it has been reported that H_2_O_2_ reacts with biomolecules at a slow rate and low micromolar concentrations of H_2_O_2_ are not sufficient to damage nucleic acids (Dizdaroglu et al., 1991; Wijeratne et al., 2005). The effect of prodigiosin and Prd/Cu(II) on dsDNA cleavage was reduced in response to the addition of tert-butanol (2 M) and NAC (10 mM) as hydroxyl radical and ROS scavengers, respectively. This suggests that in addition to H_2_O_2_, generation of other ROS molecules, such as superoxide and hydroxyl radicals known to be more reactive with nucleic acid bases, also contributed to nucleic acid degradation by prodigiosin (Dizdaroglu et al., 2002; Dizdaroglu and Jaruga, 2012).

Copper is known to facilitate oxidation of prodigiosin, thereby inducing cleavage of dsDNA (Melvin et al., 2000). Consistent with this, degradation of dsDNA in the presence of prodigiosin was enhanced through increasing concentrations of copper. The prodigiosin concentration required to cleave dsDNA was lowered at least 5 times in the presence of equal concentrations of copper. It has been known that copper (II) oxidizes H_2_O_2_ to superoxide and further reactions between reduced copper (I) and excess H_2_O_2_ form hydroxyl radicals (Pham et al., 2013). Further experiments are required to elucidate the exact DNA cleavage mechanism by prodigiosin.

**Figure 9:**
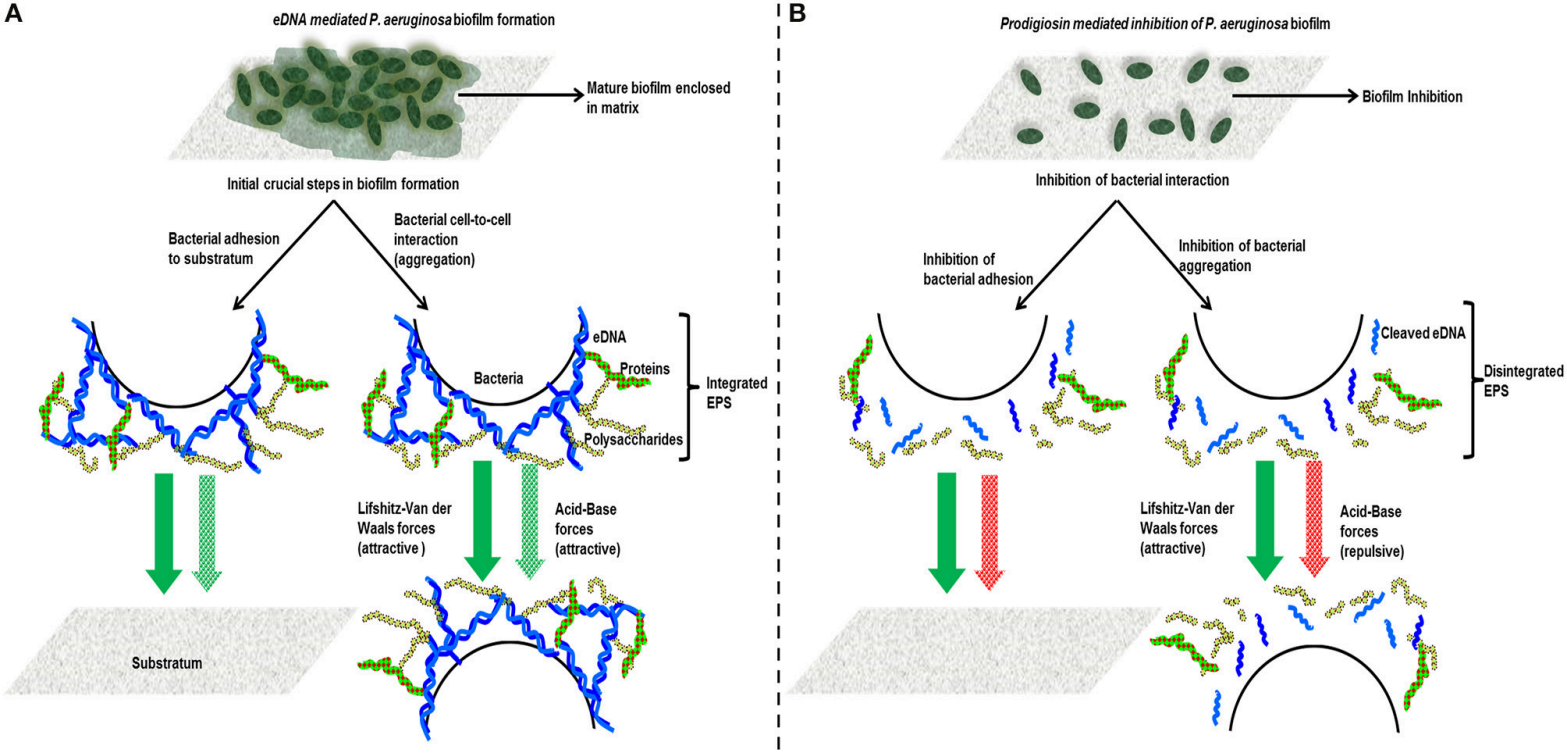
**Represents the schematic showing the role of eDNA in facilitating attractive physico-chemical interactions (Vander Waals and acid-base forces) that drives initial bacterial interaction (adhesion and aggregation) and biofilm formation. (A)** Prodigiosin cleaves eDNA and disintegrates EPS on bacterial cell surface thus inhibits acid-base interaction forces responsible for bacterial adhesion aggregation and biofilm formation **(B)**.

Contact angle measurements revealed that *P. aeruginosa* PA14 cells treated with prodigiosin undergone significant modulation in its cell surface hydrophobicity (Table 1) similar to the modulation encountered by DNase I treated *P. aeruginosa* PA14 as previously reported (Das et al., 2013a). This result confirms that cleaving of extracellular DNA attached to the *P. aeruginosa* cell surface is primarily responsible for inducing modulation in cell surface hydrophobicity. Surface energy values of prodigiosin treated cells are significantly different compared with untreated wild-type cells. Subsequent calculations of Gibbs free energy of interaction revealed that prodigiosin treated cells have less favorable interactions between cells and between cells and substratum surfaces (Figure 4). This result indicates that non-specific physico-chemical forces that include Vander Waals and acid-base and hydrophobic interactions forces originating from molecules present on interacting surfaces (van Oss, 1995; Sharma and Hanumantha Rao, 2003) are responsible to facilitate bacterial interactions (Hermansson, 1999). In the case of bacterial cells, biomolecules such as eDNA, proteins and polysaccharides anchor onto cell surfaces and extend up to hundreds of nanometres to facilitate physico-chemical forces, cross-linking between molecules and consequently promote bacterial adhesion and aggregation (Boks et al., 2008). Previous reports also suggest that eDNA act as a scaffold for biofilm by integrating EPS (Das et al., 2013b), in this study eDNA anchored to *P. aeruginosa* cell surface facilitates acid-base interaction forces to drive bacterial adhesion and aggregation. Bacterial adhesion and aggregation are the initial crucial step in the subsequent bacterial colonization and development of mature biofilm. Prodigiosin disarms the favorable physico-chemical forces by cleaving eDNA and subsequently disintegrate the EPS and thus impairs bacterial adhesion, aggregation and biofilm formation as proved using CLSM (Figure 6). Figure 9 represents the schematic of prodigiosin mediated eDNA cleaving and inhibition of bacterial interaction and biofilm formation. Prodigiosin-mediated eDNA removal was facilitated in the presence of copper, likely playing a role in disruption of established biofilms.

The concentration of prodigiosin required to inhibit biofilm formation of *P. aeruginosa* was within the range of 100–500 μM, which is at least five times higher than the reported concentrations required to induce apoptosis in human cancer cell lines (da Silva Melo et al., 2000; Montaner et al., 2000; Montaner and Perez-Tomas, 2001) and larger than the concentration of Streptorubin B to inhibit biofilm formation (Suzuki et al., 2015). Prodigiosin induces apoptotic effect in cancer cell lines through regulation of apoptopic and anti-apoptotic genes (Hassankhani et al., 2014), while the prodigiosin mediated biofilm inhibition has shown to rely on the production of reactive oxygen species. The hydrogen peroxide production was detected only at high micromolar concentrations of prodigiosin, and therefore, we used high concentrations of prodigiosin to inhibit biofilm formation. On the other hand, biofilm inhibitory mechanism of Streptorubin B remains unknown (Suzuki et al., 2015) so it is not possible to compare the concentration of prodigiosin with Streptorubin B used in biofilm inhibition assays.

A connection has been proposed between the rapid evolution of resistance to chemotherapy in cancer cells and resistance to antibiotics in bacterial biofilms (Lambert et al., 2011). It has been suggested that bacterial biofilms can be used as basic model systems to understand the regulation of drug resistance in cancer cells (Lambert et al., 2011). Prodigiosin exhibits both anti-cancer activity against cancer cells with multi-drug resistance phenotype (Pandey et al., 2009) and anti-biofilm activity against *P. aeruginosa* biofilms, therefore, the connection between the drug resistance of cancer cells and biofilm communities can be partially understood by comparing the anti-cancer and anti-biofilm mechanism of prodigiosin. The common thread may be interference with interactions between cells rather than with the cells themselves.

Disintegrating biofilm matrices represents a promising approach to improve the penetration of antibiotics/bactericidal agents into the base of biofilms and promote removal of bacterial cells and subsequently control of infections. Prodigiosin and similar synthetic compounds including electron rich pyrrolylpyrromethene structures which can undergo oxidation and promote dsDNA cleavage can be added to the list of potential biofilm disruptors (Melvin et al., 2002). Prodigiosin could be used as an antibacterial coating agent on various clinical (biomaterials, medical devices) and non-clinical (water membrane filters) related substrata. Prodigiosin, which shows comparable activity with DNase I in cleaving DNA, may also be useful as a therapy in the form of aerosols to degrade eDNA and reduce the viscosity of the sputum in lungs of cystic fibrosis patients. eDNA is one major culprit that increases the viscosity of sputum and consequently causes serious obstruction of airways and blockage of easy respiration in cystic fibrosis patients (Fuchs et al., 1994).

## Author contributions

ÖK, TD, NK, and MM planned the experiments. ÖK, TD, AI, SK, KH, and JT performed the experiments. ÖK, TD, NK, and MM contributed in writing the manuscript.

### Conflict of interest statement

The authors declare that the research was conducted in the absence of any commercial or financial relationships that could be construed as a potential conflict of interest.

## Supplementary material

Supplementary Figure 1 **Analysis of extracted prodigiosin from *S. marcescens* using mass spectrometer and NMR. (A)** NSI-MS spectra of extracted prodigiosin, **(B)** MS/MS fragmentation patterns of prodigiosin and **(C)** ^1^H-NMR peaks of prodigiosin.

Supplementary Figure 2 **Growth curve analysis of *P. aeruginosa* PA14 cultured in LB_10_ in the presence of increasing concentrations of prodigiosin and prodigiosin/Cu(II) complex**. Error bars represents standard deviations from the mean (*n* = 3) (*P* < 0.01).

Supplementary Table 1 **Effect of increasing concentrations of prodigiosin on *P. aeruginosa* PA14 biofilms grown on polystyrene substratum**. “±” represents standard deviations from the mean (*n* = 3). Bold indicate statistically significant (*P* < 0.01) differences between prodigiosin treated and non-treated cultures.
