# Supplementary figures and images for: Serratia Secondary Metabolite Prodigiosin Inhibits Pseudomonas aeruginosa Biofilm Development by Producing Reactive Oxygen Species that Damage Biological Molecules
Source: Front Microbiol. 2016 Jun 27;7:972. doi: 10.3389/fmicb.2016.00972 (PMC4922266; doi:10.3389/fmicb.2016.00972)

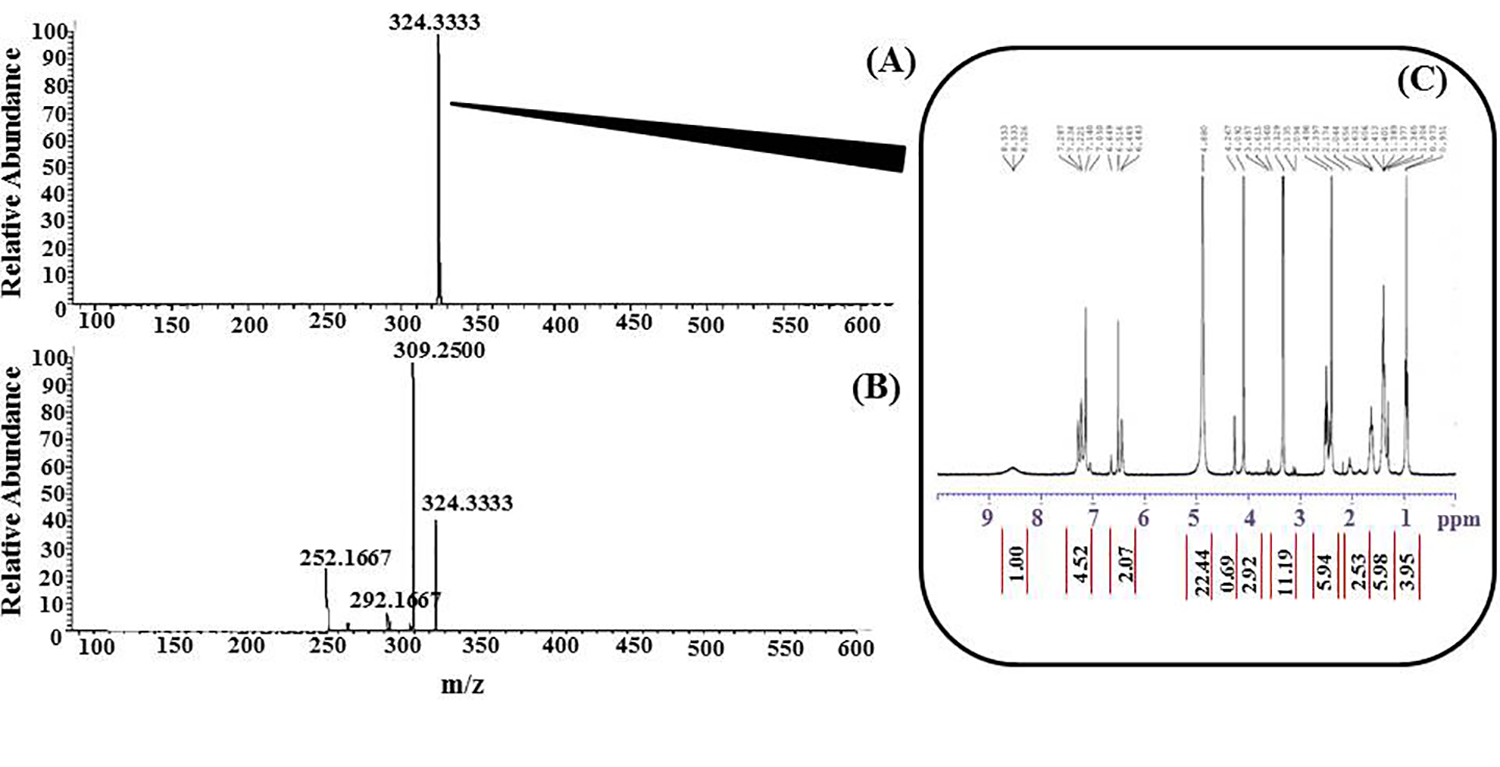

Supplement: Supplementary Figure 1 — Analysis of extracted prodigiosin from S. marcescens using mass spectrometer and NMR. (A) NSI-MS spectra of extracted prodigiosin, (B) MS/MS fragmentation patterns of prodigiosin and (C) 1H-NMR peaks of prodigiosin. [file Image1.TIF]

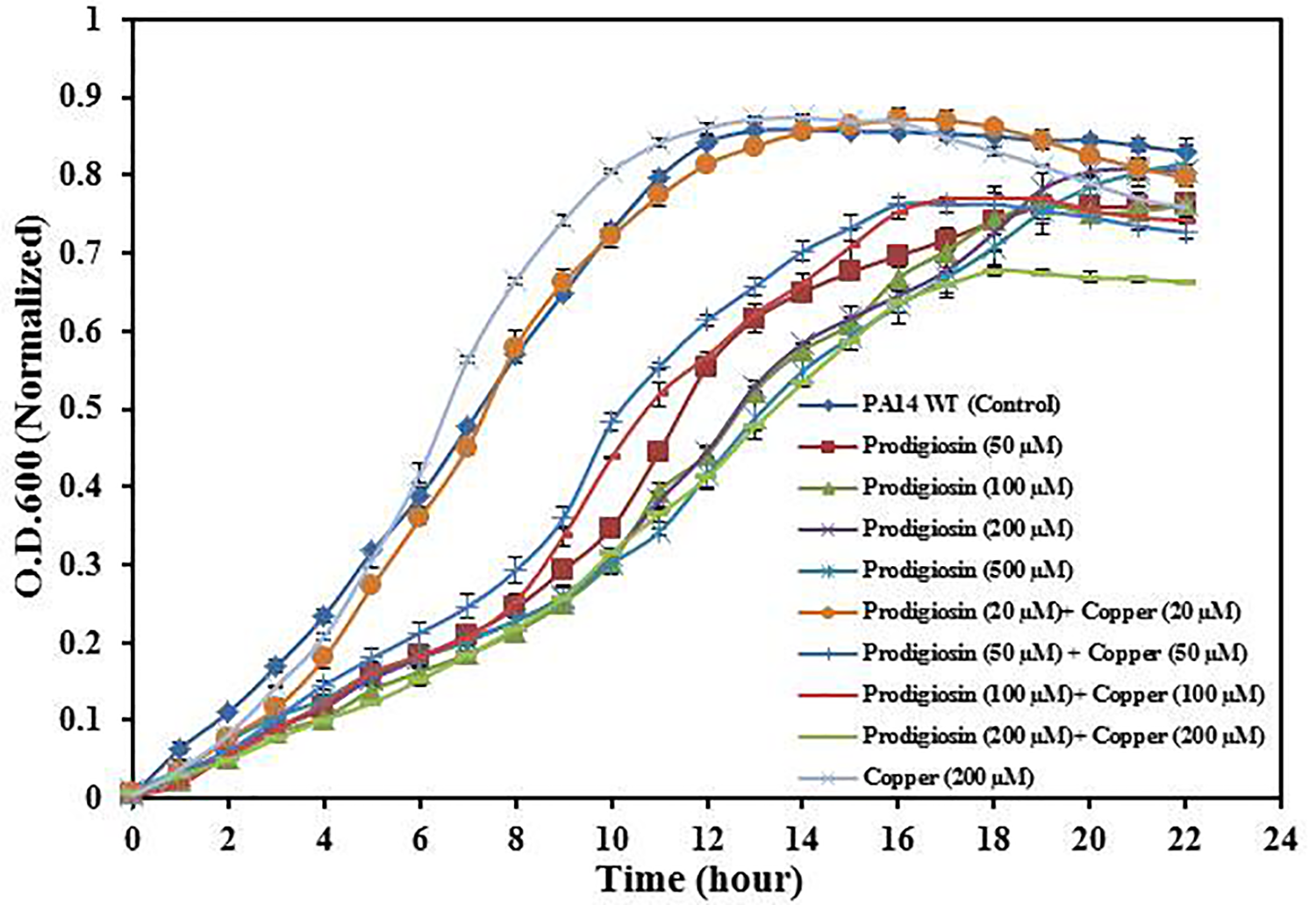

Supplement: Supplementary Figure 2 — Growth curve analysis of P. aeruginosa PA14 cultured in LB10 in the presence of increasing concentrations of prodigiosin and prodigiosin/Cu(II) complex. Error bars represents standard deviations from the mean (n = 3) (P < 0.01). [file Image2.tif]
