# Supplementary material for: Serratia Secondary Metabolite Prodigiosin Inhibits Pseudomonas aeruginosa Biofilm Development by Producing Reactive Oxygen Species that Damage Biological Molecules
Source: Front Microbiol. 2016 Jun 27;7:972. doi: 10.3389/fmicb.2016.00972 (PMC4922266; doi:10.3389/fmicb.2016.00972)
Supplement: Supplementary Table 1 — Effect of increasing concentrations of prodigiosin on P. aeruginosa PA14 biofilms grown on polystyrene substratum. “±” represents standard deviations from the mean (n = 3). Bold indicate statistically significant (P < 0.01) differences between prodigiosin treated and non-treated cultures. [file Table1.PDF]

Supplementary Table 1. Effect of increasing concentrations of prodigiosin on P. aeruginosa PA14 biofilms grown on polystyrene substratum. "±" represents standard deviations from the mean (n=3). Bold indicate statistically significant (*P*<0.01) differences between prodigiosin treated and non-treated cultures.

	**Non-established Biofilms**				**Pre-established Biofilms**	
	**Wild-type**	**Wild-type + 100 µM Prodigiosin**	**Wild-type + 200 µM Prodigiosin**	**Wild-type + 500 µM Prodigiosin**	**Wild-type**	**Wild-type + 500 µM Prodigiosin**
**Thickness**	3.9 ± 0.5	3.3 ± 0.5	3.5 ± 0.3	1.5 ± 0.2	4.1 ± 0.4	2.1 ± 0.1
**Biovolume ($\mu m^3/\mu m^2$)**	1.3 ± 0.1	0.5 ±0.07	0.2 ± 0.07	0.06 ± 0.01	1.1 ± 0.5	0.2 ± 0.1
**% live biofilm biovolume ($\mu m^3/\mu m^2$)**	37.1 ± 9.7	43.8 ± 4.1	72.0 ± 10.8	64.2 ± 7.8	32.1 ± 11.7	61.7 ± 8.1
**% dead biofilm biovolume ($\mu m^3/\mu m^2$)**	62.9 ±9.7	56.2 ± 4.1	28.0 ± 10.8	35.8 ± 7.8	67.9 ± 11.7	38.3 ± 8.1
**% coverage at substratum**	43.2 ± 6.6	13.8 ± 4.7	10.0 ± 2.0	3.5 ± 1.0	43.5 ± 12.1	6.9 ± 1.7
**Microcolony size (μm^2)**	3.1 ± 1.1	1.5 ± 0.3	0.6 ± 0.1	0.7 ± 0.1	2.3 ± 0.9	0.8 ± 0.2
