# Supplementary material for: Serratia Secondary Metabolite Prodigiosin Inhibits Pseudomonas aeruginosa Biofilm Development by Producing Reactive Oxygen Species that Damage Biological Molecules
Source: Front Microbiol. 2016 Jun 27;7:972. doi: 10.3389/fmicb.2016.00972 (PMC4922266; doi:10.3389/fmicb.2016.00972)
Supplement: Supplementary file 4 [file DataSheet1.PDF]

Isolation and characterization of prodigiosin from S. *marcescens*

Prodigiosin was extracted and purified from *S. marcescens* as described in materials and methods and analysed by high resolution NSI-MS (Suppl. Fig. 1A). A major peak had molecular weight of 323.3333 (m/z 324.3333, $[M+H^+]$) was identified as prodigiosin by direct ionisation of the pigment. Fragmentation patterns of the prodigiosin were analyzed through tandem mass spectrometry (MS/MS) (Suppl. Fig. 1B) and results were compared with published data (26). Further confirmation of the prodigiosin was performed by ^{1}H-NMR spectroscopy (Supp. Fig. 1C). The pigment was re-suspended in DMSO and methanol, separately, to use in microbiological and analytical tests, respectively.
